# Statistical process control within the Six Sigma DMAIC framework for monitoring medication dispensing errors in hospital pharmacies: a Saudi case study

**DOI:** 10.3389/fphar.2026.1895605

**Published:** 2026-09-29

**Authors:** Salma Elhag, Amal Albiladi, Mona Saeed, Amnah Alsulami, Layan Althobaiti

**Affiliations:** King Abdulaziz University , Faculty of Computing & Information Technology , Department of Information Systems, Jeddah, Saudi Arabia

**Keywords:** CTGAN, DMAIC, healthcare quality, hospital pharmacy, medication errors, Saudi Arabia, six sigma, statistical process control

## Abstract

This study employed the Six Sigma DMAIC methodology to monitor and analyze medication dispensing errors in hospital pharmacies in Saudi Arabia, with a focus on Mecca and Jeddah cities. Using aggregated data from the Ministry of Health (MOH) Statistical Yearbook 2024, a synthetic dataset was generated through a Conditional Tabular Generative Adversarial Network (CTGAN) to enable the Statistical Process Control (SPC) analysis. The p-chart application demonstrated how process stability can be evaluated within a synthetic healthcare dataset, while the calculated sigma level (2.49) represents the performance characteristics of the generated data rather than actual hospital pharmacy performance. Pareto analysis illustrated how dominant error categories can be prioritized within a synthetic dataset generated under predefined assumptions. Based on these findings, several improvement strategies were proposed, particularly targeting dispensing errors within pharmacy operations. However, due to the use of synthetic data, the improvement and control phases remain conceptual. The generated synthetic dataset enabled demonstration of Statistical Process Control (SPC) techniques within the Six Sigma DMAIC framework. The p-chart indicated statistical stability within the generated data, while Pareto analysis illustrated the prioritization of error categories under predefined synthetic distributions. Because all analyses were conducted using CTGAN-generated synthetic records, the findings should be interpreted as a methodological demonstration rather than as evidence of actual medication error performance in Saudi hospital pharmacies.

## 1 Introduction

Ensuring the safe use of medications remains one of the most persistent challenges in modern healthcare. Medication errors are a leading cause of preventable patient harm in healthcare systems worldwide, posing significant risks to patient safety and creating a substantial burden on healthcare resources (Panagioti et al., 2023). Dispensing errors remain a serious concern because they occur at a critical stage between prescribing and patient use. This makes dispensing safety an important concern not only from a clinical perspective, but also from an operational and economic one.

In Saudi Arabia, improving healthcare quality is one of the main goals of Vision 2030. Several initiatives have been introduced to support this direction, including efforts to improve medication dispensing services. For example, the Wasfaty service has helped make the dispensing process more efficient and has reduced errors associated with handwritten prescriptions. Medication tracking services have also been introduced to improve efficiency and reduce unnecessary financial waste. However, despite this progress, there is still a need for statistical approaches that can monitor dispensing processes in hospitals and support continuous quality improvement.

This study addresses that need by applying Statistical Process Control (SPC) and Six Sigma to the monitoring of medication dispensing errors. The study focuses on process stability and variation, with the aim of detecting deviations at an early stage and supporting safer and more reliable dispensing practices. In this way, the study seeks to contribute to quality improvement in hospital pharmacy services.

The study focuses on the Makkah and Jeddah regions, which are among the most populated areas in Saudi Arabia and represent major centers of healthcare demand. Because access to real institutional clinical data is limited and such data is highly sensitive, this study adopts an alternative methodological approach based on artificial intelligence. Specifically, the Conditional Tabular Generative Adversarial Network (CTGAN) is used to generate realistic synthetic data based on the 2024 Ministry of Health Statistical Yearbook (Ministry of Health, 2020). This allows the study to build an analytical setting that reflects real-world patterns and to examine the usefulness of SPC and Six Sigma tools in the context of medication dispensing quality.

The use of synthetic data generation methods has become increasingly important in healthcare quality research because access to patient-level data is often restricted by privacy, ethical, and regulatory requirements. Traditional simulation approaches such as Monte Carlo simulation and discrete event simulation have been widely applied in healthcare operations; however, these methods typically require detailed process maps, event-flow structures, transition probabilities, and operational parameters that were not available from the aggregated Ministry of Health (MOH) statistics used in this study. In contrast, Conditional Tabular Generative Adversarial Networks (CTGAN) are designed to learn complex relationships among mixed continuous and categorical variables directly from tabular datasets and generate statistically similar synthetic records. Therefore, CTGAN was selected as a pragmatic approach for expanding aggregated healthcare indicators into a structured dataset suitable for Statistical Process Control (SPC) analysis while preserving data privacy. Nevertheless, synthetic data generated through CTGAN remain dependent on the quality and assumptions of the source data and should be interpreted as a methodological approximation rather than a direct representation of real hospital pharmacy performance.

Medication errors represent a global challenge to healthcare sector because of their large impact on the patient safety by increasing morbidity and mortality, and rising healthcare costs by extended hospital stays, readmissions, additional treatments, resources waste, and financial compensation for some cases. These errors may happen in multiple processes including prescribing, dispensing, and administration for medicine. An analytical study in Saudi Arabia highlighted that nearly 44.4% of the medication errors happened during the clinical practice, while dispensing errors is nearly 28.2% of all incidents, finding that a substantial proportion of errors arise at the pharmacy medicine dispensing process activities (Almalki et al., 2021).

A national review of medication safety in Saudi Arabia reported that errors occur across all stages of the medication-use process, including dispensing (Alghamdi, 2025). Although dispensing errors are often under-reported, they represent a significant risk due to their direct impact on patient safety (Alghamdi, 2025). At the regional level, a retrospective cross-sectional study analyzing 19,645 medication error reports from nine hospitals in the Al-Baha region between 2023 and 2024 found that prescribing errors accounted for 94.8% of all reported errors, with wrong or missed doses being the most common type, while incomplete documentation was the lowest. Although this figure reflects the prevalence of prescribing issues in reported data, it also highlights that the reliance on self-reported systems may not capture dispensing errors accurately (Alghamdi, 2026).

A systematic review of medication errors across Saudi Arabian hospital settings analyzed 28 studies using the PRISMA-P methodology and identified wrong doses, improper doses, and prescribing errors as the most frequently reported types (Tobaiqy and MacLure, 2024). The review recommended future research focused on longitudinal and multi-center studies, standardization of study designs, and greater integration of technological solutions (Tobaiqy and MacLure, 2024). Similarly, a systematic review examining the nature of dispensing errors in hospital pharmacies, covering 15 studies from 2000 to 2015, found error rates ranging widely from 0.015% to 33.5%, with wrong medicine, wrong strength, and wrong dosage form being the most common categories (Aldhwaihi et al., 2016). Heavy workload, insufficient staffing, confusing drug names, and inexperienced workers were identified as primary contributing factors (Aldhwaihi et al., 2016). The wide variation in error rates across studies reflects the absence of standardized monitoring methods, a gap that structured statistical approaches can directly address (Tobaiqy and MacLure, 2024; Aldhwaihi et al., 2016).

Understanding the operational context of dispensing in Saudi hospitals is essential for identifying where errors occur and where monitoring is most needed. A national survey of dispensing and administration practices in 21 hospitals in Jeddah, Saudi Arabia, found that 76.2% of hospitals relied on centralized pharmacy systems and that the technician-fills/pharmacist-checks model was used by 76.2% of facilities (Altyar et al., 2020). However, only 28.6% reported using automated dispensing cabinets and 19.0% used barcode medication administration systems (Altyar et al., 2020). The study was descriptive in nature and did not include continuous performance monitoring or real-time error tracking. Evidence from other healthcare contexts similarly highlights the role of dispensing system design in prevention of error. A comparative analysis of dispensing systems in Brazil, for example, showed that the unit-dose system was associated with a reduction in errors from 31% to 13%, though adoption remained low due to cost and training requirements (Anacleto et al., 2005). These findings confirm that the dispensing system itself is a significant determinant of error risk and that monitoring frameworks must account for system-specific characteristics.

Several studies have examined the role of technology in reducing medication dispensing errors. A review of technology-based interventions found that computerized physician order entry systems reduced errors by 55%–83%, barcode systems reduced administration errors by 54%–87%, and automated dispensing systems cut dispensing errors by approximately 31% (Agrawal, 2009). Within the Saudi context, a 21-month usability study on robotic pharmacy implementation reported a reduction in overall error rates and improved staff satisfaction (Momattin et al., 2021). A study at King Fahad Armed Forces Hospital implementing automated dispensing cabinets using a Six Sigma approach reported an 83% reduction in medication turnaround time and substantial cost savings (Almalki et al., 2023). A multi-center Saudi study evaluating automated dispensing cabinets found a 25.3% reduction in overall medication errors, though findings relied on self-reported data (Alshamrani et al., 2025). In critical care settings, a controlled before-and-after study found that dispensing errors dropped to zero following automated dispensing cabinet implementation, and a systematic review of pediatric interventions demonstrated that technology-based controls were 1.4 times more effective than educational or policy-based approaches alone (Tu et al., 2023; Koeck et al., 2021).

Beyond technological solutions, structured quality improvement methodologies have demonstrated substantial effectiveness in reducing medication-related errors and improving pharmacy performance. The Six Sigma DMAIC framework has been successfully implemented in hospital pharmacy settings across different healthcare systems. A multidisciplinary Six Sigma project in a Saudi university hospital outpatient pharmacy achieved a reduction in dispensing error rates from 1.00% to 0.24% and reduced inventory waste costs by 83.9% (Al Kuwaiti, 2016; Al Nemari and Waterson, 2022). Similarly, a Saudi hospital study utilizing cause-and-effect analysis, Pareto charts, and statistical process control techniques reported improvements in medication administration processes and sigma performance (Ashgar et al., 2025). Additional evidence from hospital pharmacy settings has shown that Six Sigma tools can improve medication safety outcomes, reduce process variation, and enhance operational efficiency through structured data-driven improvement initiatives. These findings demonstrate the growing applicability of DMAIC as a framework for improving medication-use processes and support its application to medication dispensing quality monitoring in Saudi healthcare settings.

At the international level, a systematic review of Lean Six Sigma applications in hospital pharmacy services reported error reductions ranging from 42% to 91% (Sallam, 2024). A prospective study from a Chinese teaching hospital combined Lean Six Sigma with Failure Mode and Effects Analysis to identify high-risk medications and achieve statistically significant reductions in dispensing errors (Chen et al., 2020). A Thai hospital case study applying the DMAIC framework achieved a 55% reduction in dispensing errors, with statistical process control charts used both to establish baseline performance and to track improvements during the control phase (Trakulsunti et al., 2022). A 14-year retrospective study from a Chinese hospital outpatient pharmacy covering over 41 million prescriptions demonstrated that sustained quality control efforts led to a 96% reduction in dispensing errors (Gao et al., 2023). A quality improvement intervention at Johns Hopkins Aramco Healthcare in Saudi Arabia, targeting the time to administration of urgent medications, reported a reduction in turnaround time from 59.7 to 40.7 min and an increase in on-time administration from 20% to 67% (Stephen et al., 2017).

Statistical Process Control (SPC) provides a structured approach to monitor process performance over time and to distinguish routine variation from changes that may indicate a real shift in performance. A systematic review of SPC applications in healthcare quality improvement demonstrated that control charts have been widely used to monitor performance and detect changes across various healthcare processes (Thor et al., 2007). The review noted, however, that SPC was sometimes applied incorrectly or only retrospectively, limiting its effectiveness as a prospective tool (Thor et al., 2007). An early application of SPC within a Continuous Quality Improvement initiative used run charts, Pareto charts, and control charts to monitor medication error rates and distinguish meaningful process changes from routine variation, providing foundational evidence of SPC feasibility in medication-related processes (Carey and Teeters, 1995). A study at Al-Herafeen Healthcare Unit in Egypt applied SPC and Total Quality Management tools including Pareto charts, control charts, and fishbone diagrams to the pharmacy dispensing process, finding that 60% of errors occurred in the pharmacy and confirming that these tools can accurately identify root causes and support ongoing monitoring (Abd El-Rasheed et al., 2020). A benchmarking study at King Hamad University Hospital in Bahrain reported a 32.5% reduction in medication incident rates after a structured benchmarking framework was applied, though benchmarking depends on retrospective data and does not enable real-time detection of process deviations, which represents a fundamental advantage of SPC-based monitoring (ElLithy et al., 2023).

The reviewed literature shows that medication dispensing errors remain a significant patient safety concern in Saudi hospital pharmacies, and that current monitoring practices are largely descriptive and retrospective in nature. While automation and structured quality improvement methodologies such as Six Sigma and Lean have shown measurable improvements in error rates, their application has been primarily focused on intervention outcomes rather than continuous process surveillance. SPC has been widely used as a quality improvement tool in healthcare, yet its direct and systematic application to monitoring medication dispensing errors in Saudi hospital pharmacy settings remains limited. The present study addresses this gap by applying SPC tools within a DMAIC framework to monitor, analyze, and improve medication dispensing error rates, aiming to demonstrate that proactive, data-driven monitoring can build a more reliable dispensing process in the Saudi healthcare context.

The contribution of this study differs from prior healthcare SPC and Six Sigma investigations in that it focuses on methodological feasibility under conditions of limited data availability. Most previous studies applied SPC and DMAIC using real operational datasets, whereas this study evaluates whether publicly available aggregated healthcare statistics can be expanded through synthetic data generation to support SPC-based quality monitoring workflows. Therefore, the contribution is methodological rather than empirical, providing a proof-of-concept framework that may support future studies when access to patient-level data is restricted.

Accordingly, the purpose of this study is not to estimate actual medication error rates or process capability within Saudi hospital pharmacies. Rather, it is to demonstrate how Statistical Process Control tools can be integrated within the Six Sigma DMAIC framework when only aggregated healthcare statistics are available. The study therefore serves as a methodological proof-of-concept using synthetic data generated from publicly available healthcare indicators.

## 2 Methodology

This study aims to apply the Six Sigma DMAIC (Define–Measure–Analyze–Improve–Control) methodology, as shown in Figure 1, to model and monitor medication errors within selected Saudi healthcare regions, specifically Mecca and Jeddah. Public statistics published in the Saudi Ministry of Health (MOH) Statistical Yearbook 2024 are used to establish the baseline performance level (Ministry of Health, 2020). As the MOH data are reported in aggregated form, a Conditional Tabular Generative Adversarial Network (CTGAN) is employed to expand the baseline into a structured synthetic dataset, as shown in Figure 2, suitable for Statistical Process Control (SPC) analysis. The generated dataset is fully synthetic and is used for methodological purposes only. It does not reflect actual hospital patient records.

**FIGURE 1:**
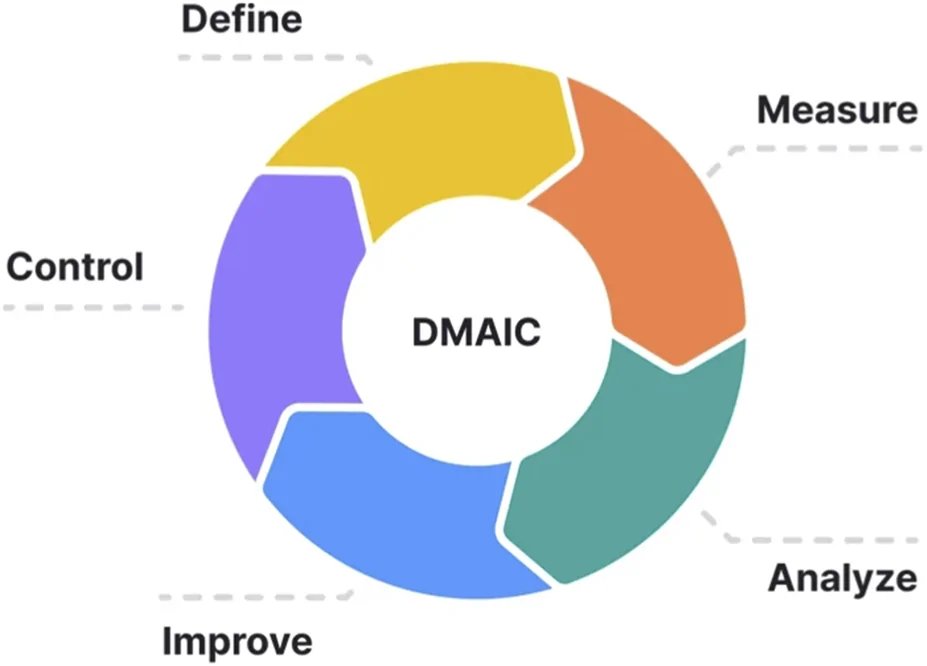
Six Sigma DMAIC methodology.

**FIGURE 2:**
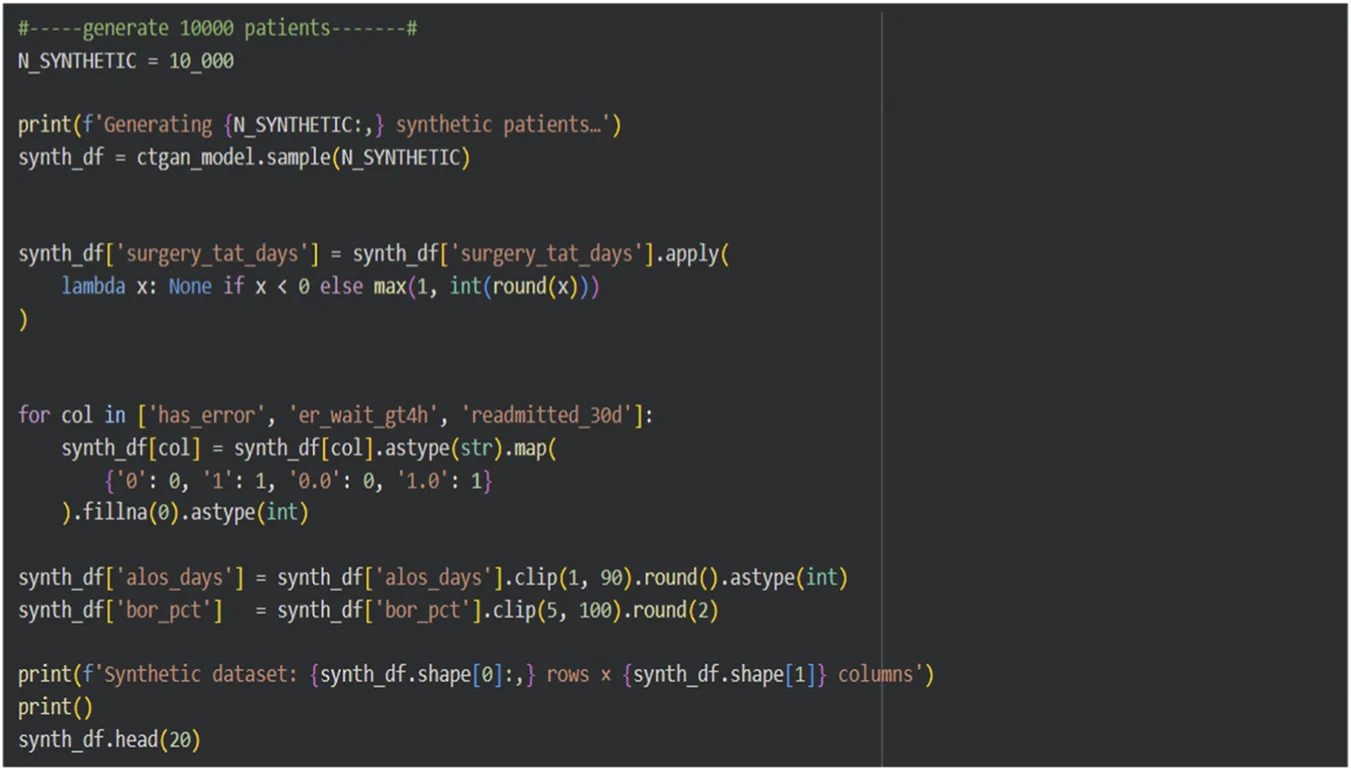
Conditional tabular generative adversarial network (CTGAN) to expand the baseline into a structured synthetic dataset.

### 2.1 CTGAN configuration and validated

First, the process starts with building the Seed Dataset, which is the foundation that CTGAN learns from. We built 2000 rows using real MoH numbers as a baseline, which consists of:

- BOR and ALOS per cluster were collected directly from Table 4-25 of the Saudi Ministry of Health Statistical Yearbook 2024.
- Admission volumes were obtained from Table 4-22 of the Saudi Ministry of Health Statistical Yearbook 2024.
- Age distribution was set using the Saudi population pyramid.
- Diagnosis groups were 12 ICD-10-chapter proxies with manuals set probabilities matching the KSA disease burden.
- Error types were set with a base rate of ∼5.8% total errors, split across 8 WHO error categories.

The error-category distributions and prevalence rates incorporated into the seed dataset were assumption-based and informed by published literature rather than derived from patient-level observations. Consequently, downstream analytical outputs may partially reflect the assumptions embedded during dataset construction. The generated results should therefore be interpreted as demonstrations of analytical procedures rather than empirical findings.

Then, define the Metadata by manually forcing the Categorical columns (region, health-cluster, age-group, gender, nationality, diagnosis_group, error_type), transform the Boolean columns (has_error, er_wait_gt4h, readmitted_30) into 0/1 integers, for numerical columns, the CTGAN uses Gaussian mixture models internally, and apply NaN handling for (surgery_tat_days) for non-surgical patients.

After that, select the Hyperparameters. The CTGAN model was configured using empirically selected hyperparameters commonly reported in tabular synthetic data generation studies. Detailed model settings, software specifications, random seed configuration, and implementation scripts are provided in Supplementary Material.

#### 2.1.1 Show more lines

Then, CTGAN needs an explicit Python list of categorical column names.

After defining columns, we use (seed_clean, discrete_columns = discrete_cols). For training data. The generator takes random noise and produces fake patient rows, the discriminator compares the fake row against a real seed row and scores how convincing it is, and after that, the generator updates its weights to produce more convincing rows. The discriminator updates its weight to get better at detecting fakes. Finally, this step repeats 300*(2000/500) = 1.200 gradient steps in total.

Finally, the model generates 10,000 rows* 14 columns of output.

### 2.2 Reproducibility measures

To facilitate reproducibility and independent verification, all model parameters, synthetic data generation procedures, validation summaries, and analysis scripts have been documented and included in the Supplementary Material. The CTGAN training configuration, random initialization seed, seed dataset construction methodology, and synthetic data fidelity assessment are reported to ensure methodological transparency.

#### 2.2.1 Synthetic data validation and fidelity assessment

To evaluate the fidelity of the generated dataset, the distributions of key variables in the synthetic data were compared with the assumptions used to construct the seed dataset. Attention was given to medication error prevalence because it served as the primary quality metric for subsequent SPC analysis. While the seed dataset was configured with an overall medication error prevalence of approximately 5.8%, the generated dataset produced an overall defect rate of 16.05%. This discrepancy suggests potential distributional drift and overrepresentation of the minority error class during synthetic data generation. Such behavior has been previously reported in generative models when learning imbalanced categorical distributions.

Consequently, the calculated defect rate, DPMO, and sigma level should be interpreted as outputs of the synthetic dataset rather than direct estimates of real-world medication error performance. The results therefore demonstrate the methodological application of SPC and Six Sigma tools on synthetic healthcare data rather than the actual performance of hospital pharmacy systems. Future research should include formal statistical validation of synthetic datasets using distribution-comparison metrics and external validation against real-world pharmacy data.

### 2.3 Statistical validation metrics

To evaluate synthetic data fidelity, multiple statistical similarity measures were computed between the seed dataset and generated dataset. These included comparisons of marginal distributions, category-frequency preservation, summary statistics (mean, standard deviation, minimum, maximum), and relative frequency errors across categorical variables. In addition, medication-error prevalence in the generated dataset was compared with the prevalence specified during seed-data construction. The objective was not to demonstrate equivalence to real-world hospital data but rather to assess whether the generated data reasonably preserved the characteristics intentionally embedded within the seed dataset.

Because no patient-level reference dataset was available, advanced validation approaches such as Kolmogorov-Smirnov testing, Wasserstein distance analysis, correlation preservation metrics, propensity-score disclosure risk assessment, and external validation against hospital pharmacy records could not be performed. Therefore, the fidelity assessment is limited to internal comparisons between the seed dataset and the generated dataset.

#### 2.3.1 Define phase

In the Define phase, the problem is framed as the need to systematically monitor medication errors as a measurable quality indicator within healthcare operations. The analysis focuses on the selected regions of Mecca and Jeddah to ensure a consistent and controlled evaluation scope.

The primary critical-to-quality (CTQ) characteristic was defined as the overall medication error rate. A broad medication-error metric was selected because the synthetic dataset was designed to represent the complete distribution of medication error categories rather than dispensing errors alone. Dispensing-related errors were subsequently selected as a focal area for improvement because they fall within the direct control of pharmacy operations and represent a practical target for intervention within the DMAIC framework. Thus, the SPC analysis evaluates overall medication error performance, while the Improve and Control phases emphasize pharmacy-specific opportunities for quality improvement.

### 2.4 Measure phase

In the Measure phase, the CTQ was quantified using the synthetic dataset generated from the MOH Statistical Yearbook 2024 baseline (Ministry of Health, 2020). The analysis was limited to records from the selected regions of Mecca and Jeddah to maintain a consistent evaluation scope. A binary defect variable was created to distinguish medication error events from non-error outcomes. Records labeled as medication errors were treated as defects, while those labeled “No Error” were considered non-defective observations. To enable Statistical Process Control (SPC) analysis, the filtered dataset was divided into ten equal subgroups, each consisting of 1,000 observations. To demonstrate SPC calculations, the generated dataset was divided into ten equal subgroups of 1,000 observations each. The subgroup structure does not correspond to a real sampling design, audit schedule, pharmacy shift structure, or operational monitoring system. Instead, fixed-size subgroups were used solely to illustrate SPC calculations. Consequently, the subgroup proportions should not be interpreted as estimates derived from operational healthcare processes.

In practical SPC applications, subgroup size is typically determined by process characteristics, data collection frequency, and desired chart sensitivity. Therefore, the resulting control limits should be interpreted within the context of this methodological example rather than as operational limits for a real pharmacy process.

For each subgroup, the number of defects was calculated, and the medication error rate was computed as the proportion of defective records within that subgroup. These subgroup proportions were used in the p-chart and Six Sigma analysis.

#### 2.4.1 Analyze phase

In the Analyze phase, the objective was to examine process stability, evaluate overall performance capability, and identify the main contributors to medication errors within the selected regions of Mecca and Jeddah. The stability of the medication error process was first assessed using a p-chart constructed from 10 subgroups, each consisting of 1,000 observations.

The average error rate (p̄) was calculated as 0.1605 and used as the central line of the control chart. Based on the subgroup size (n = 1,000), the upper and lower control limits were determined as 0.1953 and 0.1257, respectively, as shown in Figure 3. The P-chart was based on basic assumptions to ensure the accuracy of the results, including the independence of the observations, the large sample size which allows for normal approximation, and considering each patient record as a single opportunity for evaluation. These assumptions cannot be independently verified because the records were algorithmically generated rather than collected from a naturally occurring healthcare process. Therefore, the p-chart should be regarded as a methodological illustration of SPC implementation under synthetic-data conditions rather than evidence of true process behavior.

**FIGURE 3:**
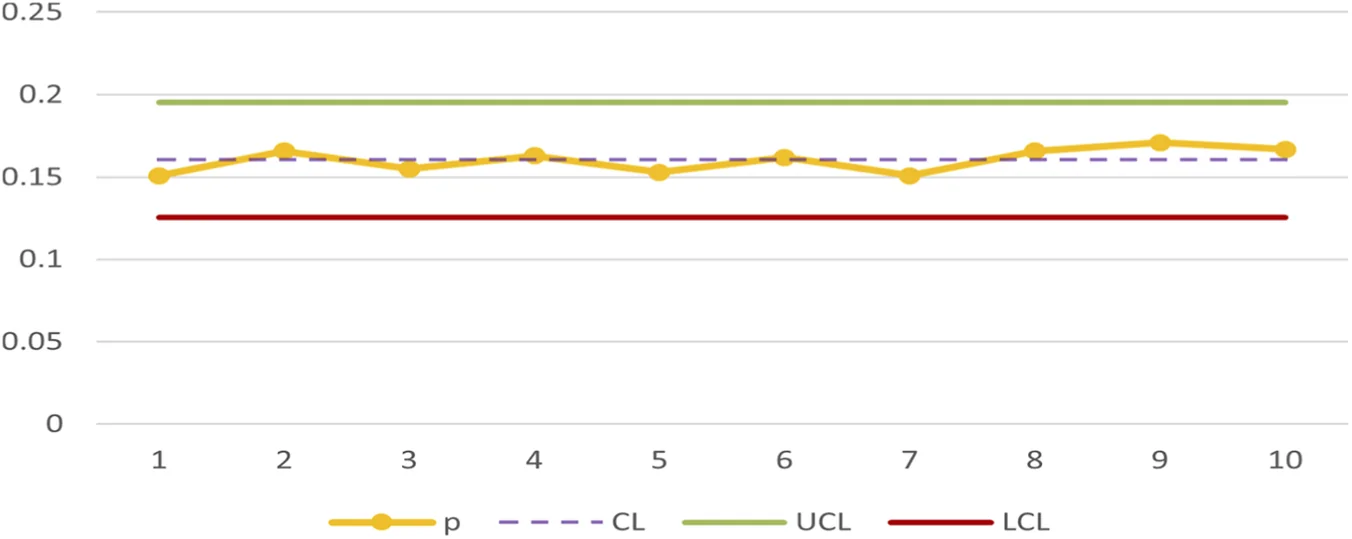
P-chart for medication error rate in Mecca and jeddah**.**

The subgroup proportions ranged between 0.151 and 0.171, and all values remained within the calculated control limits. No points exceeded the upper control limit or fell below the lower control limit. This indicates that the observed variation is due to common-cause factors rather than special-cause variation. Therefore, the medication error process in Mecca and Jeddah can be considered statistically stable under the current conditions.

Following the stability assessment, overall process capability was evaluated using Six Sigma metrics. A total of 1,605 defects were identified across 10,000 observations, resulting in an overall defect rate of 0.1605. Assuming one opportunity for error per record, the Defects Per Million Opportunities (DPMO) were calculated at approximately 160,500.

The Defects Per Million Opportunities (DPMO) value was calculated by dividing the number of defective observations by the total number of observations and multiplying by one million, assuming one medication-error opportunity per record. Because both the defect count and defect rate originate from the synthetic dataset, the resulting sigma level should be interpreted as a characteristic of the generated data rather than a measure of actual pharmacy process capability. Therefore, Figure 4 is intended to illustrate the calculation conceptually and should not be interpreted as a formal process capability analysis.

**FIGURE 4:**
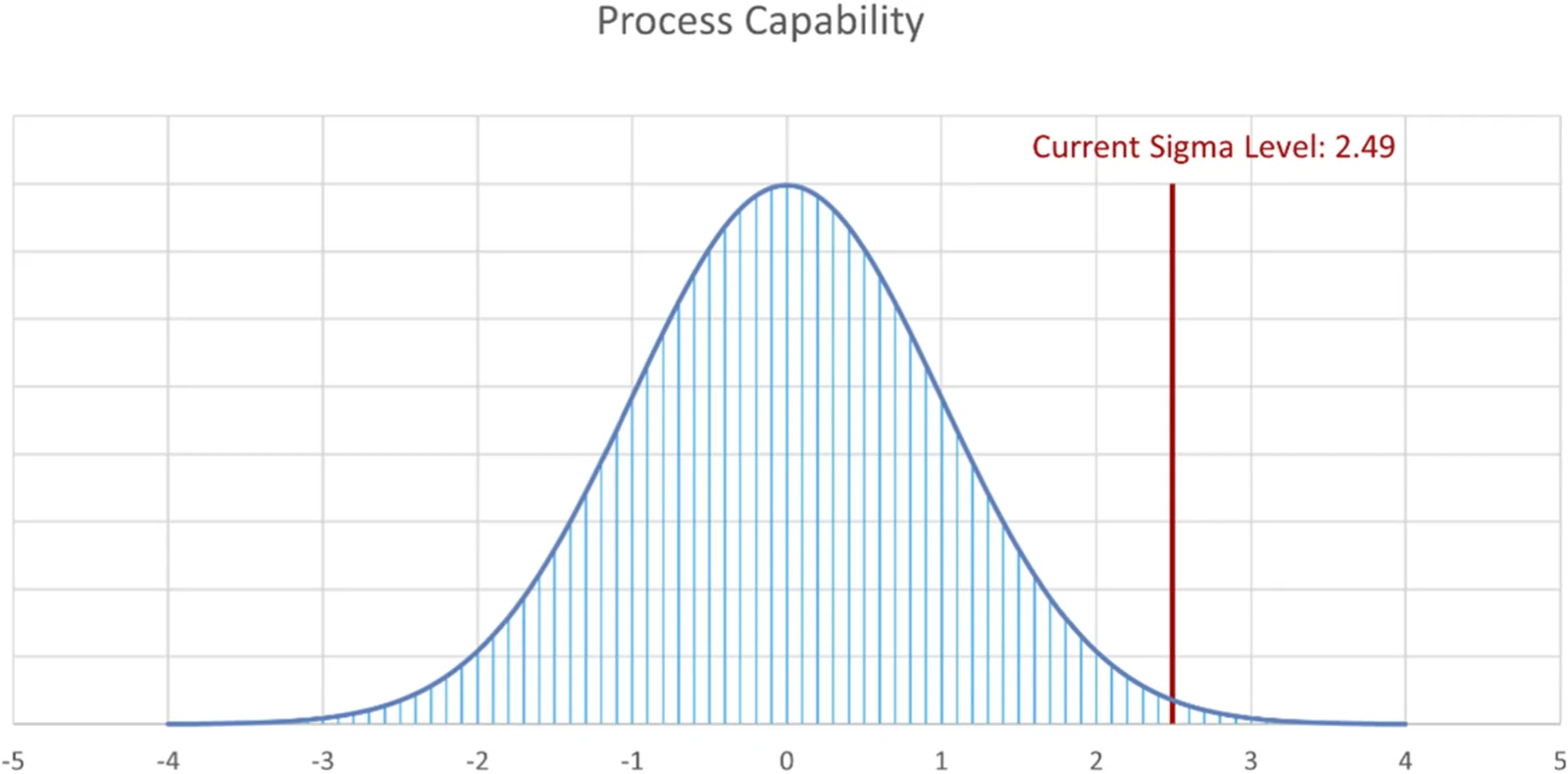
Conceptual illustration of sigma-level position based on the generated synthetic dataset.

Based on the standard Six Sigma conversion, this corresponds to a sigma level of 2.49. Within the generated dataset, the sigma level corresponds to the defect prevalence produced by the synthetic data generation process. Therefore, it should not be interpreted as an estimate of the capability of any actual hospital pharmacy system. Its primary purpose is to demonstrate the computational application of Six Sigma metrics.

The sigma position is visually illustrated in Figure 4.

To further understand the distribution of errors, a Pareto analysis was conducted to identify the most significant contributors to total medication errors, as shown in Figure 5.

**FIGURE 5:**
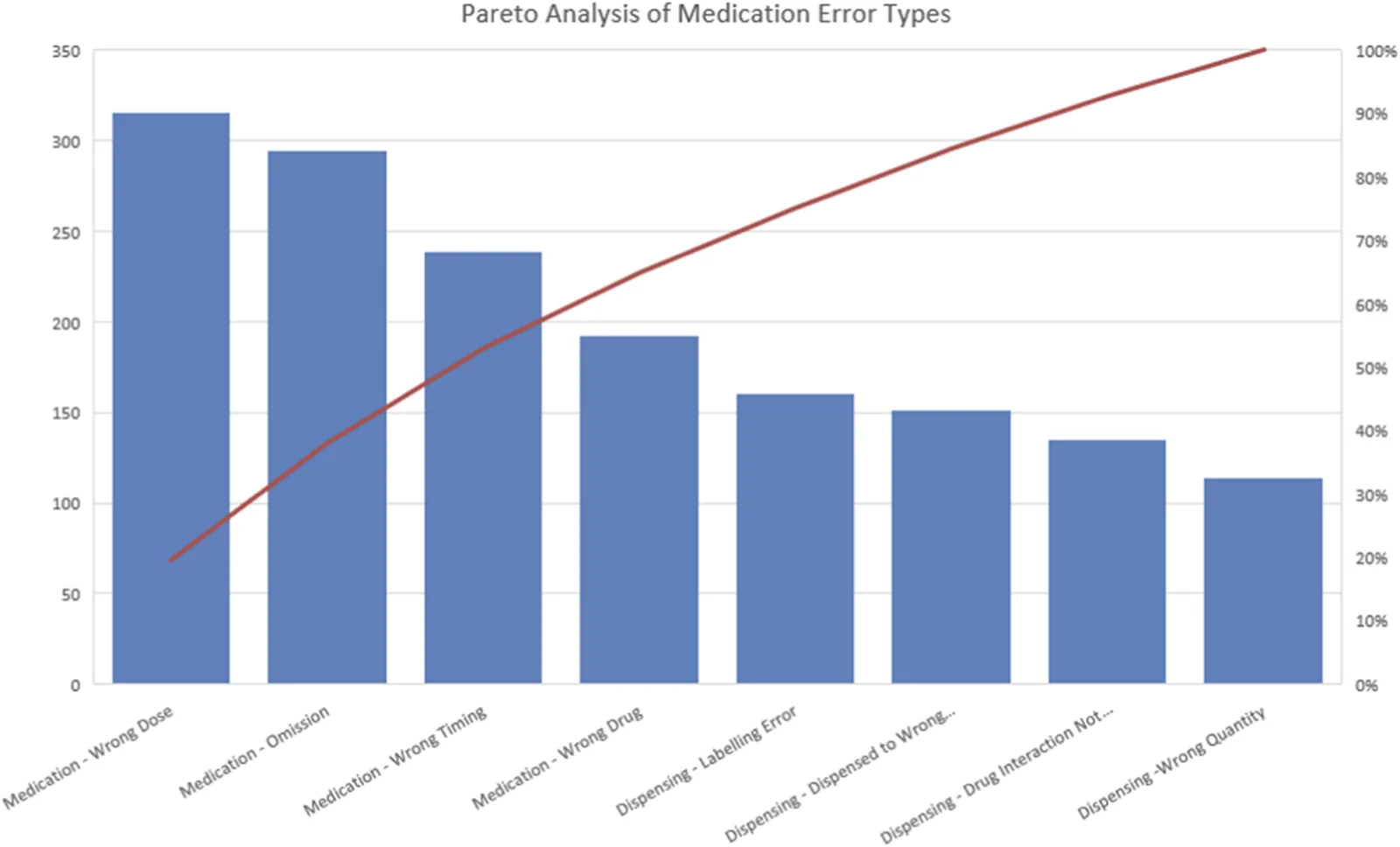
Pareto analysis of medication error types.

The Pareto analysis indicates that a limited number of error categories account for a substantial proportion of total defects within the generated dataset. Medication–Wrong Dose (316 cases), Medication–Omission (295 cases), and Medication–Wrong Timing (239 cases) were identified as the most frequently occurring error categories. Together, these categories represent more than half of the total 1,605 errors generated.

However, it is important to note that these frequencies reflect the predefined probability distributions and assumptions used during synthetic dataset construction and CTGAN generation. Consequently, the findings should not be interpreted as observed medication error distributions in actual hospital pharmacies. Rather, the Pareto analysis serves as a methodological demonstration of how Six Sigma tools can be used to prioritize quality improvement efforts when error data is available.

To explore potential contributors to dispensing-related errors, a conceptual cause-and-effect (Fishbone) analysis was developed, as illustrated in Figure 6. The diagram was not derived directly from the synthetic dataset because the generated data does not contain variables representing operational factors such as workload, staffing levels, communication quality, or information system performance. Instead, the proposed causes were identified from the medication safety literature and established pharmacy quality-management practices. Therefore, the Fishbone diagram should be interpreted as a theoretical quality-improvement exercise intended to support future root-cause investigations using real-world operational data.

**FIGURE 6:**
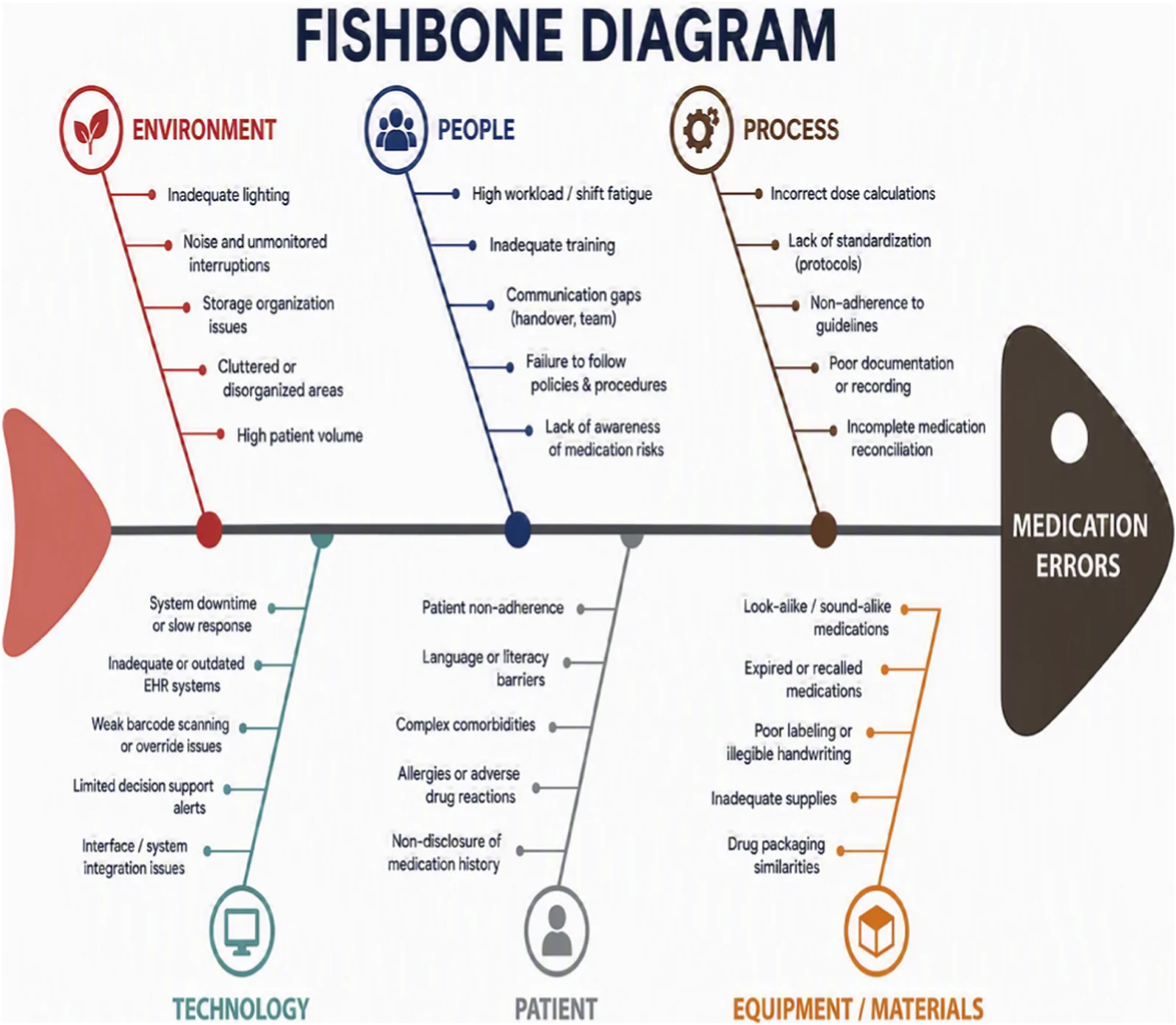
Fishbone Diagram to analyze the root cause of medication dispensing errors.

#### 2.4.2 Improve phase

The Improve phase was not executed in a real healthcare setting and no intervention outcomes were measured. Accordingly, the proposed actions represent hypothetical quality-improvement strategies derived from the literature and the conceptual root-cause analysis rather than validated process improvements.

While the dispensing errors were less frequent than other medication error types, we have selected them for improvement because they are directly controllable within the pharmacy operations and align with the scope of this study. Furthermore, while SPC analysis indicated that the medication error process is statistically stable, the observed sigma level (2.49) remains below desirable performance standards, which justifies the need for structured improvement efforts. Therefore, the following improvement interventions are proposed conceptually based on our review and observation, without implementation due to data limitations. The proposed interventions were selected based on the combined findings of the Pareto analysis and the conceptual fishbone analysis. The Pareto analysis indicated that wrong dose, omission, and wrong timing represented the most frequent error categories within the generated dataset, while the fishbone analysis highlighted potential contributors related to verification processes, communication practices, technology utilization, workflow design, and staff training. Consequently, the proposed interventions prioritize strengthening medication verification, reducing human error, improving process standardization, and enhancing decision support capabilities. The recommendations are presented conceptually and are not ranked by effectiveness because no implementation or outcome evaluation was performed in this study.

1. Digital Dispensing Verification through Sehhaty Integration

Integration of the pharmacy information systems with the Sehhaty application may enable the automated verification of patient identity, prescription information, dose, and timing prior to the dispensing of medication (Leaning Business Services, 2026). This strategy aims to address dispensing errors such as “wrong patient,” “wrong dose,” “wrong timing,” and “wrong quantity.” In the Saudi Arabian setting, it is feasible to utilize the existing digital infrastructure of the MOH and the Digital Twin functionality to develop patient-centric profiles that include prescription history, patient demographics, and risk factors. This would minimize the need for manual verification and improve the accuracy of medication dispensing in hospitals and clinics by reducing human error.

2. Barcode-Assisted Medication Dispensing

Implementation of the barcode scanning process for a wider range of medications, excluding only high-risk and controlled substances, may ensure that every medication is accurately linked to its corresponding approved electronic prescription. This strategy aims to correct “wrong drug,” “wrong dose,” “mislabeling,” and “wrong quantity” errors. In the Saudi Arabian setting, this strategy may utilize the existing MOH processes and extend the standardized barcode verification process to secondary hospitals and community pharmacies.

3. Kaizen Continuous Improvement Initiatives

Using the Kaizen approach requires the implementation of short-cycle reviews of the dispensing process, during which pharmacy personnel can identify small areas of inefficiency or potential sources of error and make corresponding improvements. This approach may assist in the reduction of dispensing errors. In Saudi hospitals, personnel can have short meetings to discuss small improvements and implement them.

4. Standardized Dispensing Checklists

Use checklists to standardize pharmacists’ verification processes during dispensing can help avoid errors like mislabeling patient, dose, and quantity errors. In the Saudi environment, checklists can be incorporated into the MOH hospital work environment and associated with computerized verification systems. Training staff on how to use the checklist correctly will ensure consistency, especially for high-risk medications.

5. Pharmacist Training Programs

Training pharmacists on common error types, such as dose calculation errors, timing errors, and label verification errors, can directly contribute to a reduction in wrong dose, wrong timing, mislabeling, and wrong quantity errors. In Saudi Arabia, training programs can be conducted through MOH continuing education programs and hospital training sessions, enhancing staff capacity on safe dispensing practices.

6. High-Risk Medication Flagging

Implementation of flags for high-risk medications in pharmacy software may trigger further verification procedures prior to dispensing. This strategy aims to reduce errors of wrong dose, wrong patient, and mislabeling. In the Saudi environment, the flags can be linked with the MOH electronic prescription systems to give real-time notifications to pharmacists, improving safety for high-risk medications.

#### 2.4.3 Control phase

The Control phase is presented exclusively as a conceptual framework. Since no intervention was implemented and no post-improvement measurements were collected, the effectiveness of the proposed control mechanisms cannot be evaluated within the scope of this study.

To facilitate the continuous monitoring of the proposed improvement actions, and since the process is statistically stable but operating at a suboptimal level of performance, a combination of Statistical Process Control and Total Quality Management tools is conceptually suggested. The proposed tools are meant to facilitate continuous monitoring, reduce process variation, and provide early warnings of impending performance decline, although empirical validation is not feasible.

1. P-Charts for Medication and Dispensing Error Rates: P-charts are recommended for monitoring the proportion of medication and dispensing errors over time to identify special-cause variation and process stability.
2. Run Charts for Short-Term Trend Detection: Run charts are recommended as a complementary approach to detect trends or changes in error rates before the control limits are reached.
3. SPC-Based Regional Performance Dashboards: The establishment of a centralized dashboard under the oversight of the Saudi Ministry of Health is recommended to display SPC metrics for comparison across regions and inform managerial decisions.
4. Benchmarking Against National Performance Indicators: Benchmarking is recommended to compare the regional dispensing performance against national standards and past MOH performance targets to enable objective assessment of relative process capability.
5. Standardized Process Control Plans: Process control plans are recommended to formally identify key dispensing processes, points of verification associated with electronic prescriptions accessible through the Sehhaty portal, and related monitoring responsibilities.
6. Periodic Internal Audits and Quality Reviews: Periodic audits and quality reviews are recommended as a complementary approach to TQM to evaluate compliance with standardized dispensing practices and identify opportunities for C&PA.

Due to the use of synthetic data in this paper, the Improve and Control phases are presented as a conceptual design. These phases are proposed based on the statistical analysis but have not been implemented or measured in real data.

## 3 Limitations

This study is subject to several limitations, primarily related to data generation and representativeness. First, the dataset used for analysis is fully synthetic, generated using CTGAN based on aggregated statistics from the MOH Statistical Yearbook 2024. Although this approach provides methodological application and simulation of realistic patterns, it does not reflect the actual data or real hospital pharmacy processes.

Second, the lack of a real sampling frame limits the ability to assess the representativeness of the dataset. Since the data are derived from aggregated national statistics rather than sampled observations from specific hospitals or pharmacies, there is no clear indication of how well the synthetic dataset captures variations across different healthcare settings, regions, or patient populations.

Third, the generalizability of the results is constrained. The results, including process stability and sigma level, are dependent on the assumptions embedded in the synthetic data generation process, such as predefined error rates and distributions. As a result, the conclusions cannot be directly generalized to actual hospital pharmacies in Saudi Arabia without empirical validation using real-world data.

Although Supplementary Material provide sufficient information to reconstruct the synthetic data generation process, the generated dataset remains dependent on assumptions embedded within the seed dataset and CTGAN modeling framework. Therefore, reproducibility of the synthetic results does not necessarily imply validity with respect to real-world hospital pharmacy performance.

Finally, the Improve and Control phases are conceptual and have not been implemented or tested in practice. Therefore, the effectiveness of the proposed interventions remains unverified, and further empirical studies are required to evaluate their real-world impact.

## 4 Discussion

The synthetic dataset generated for this study exhibited statistical characteristics that produced a stable p-chart pattern under the selected SPC configuration. Because the observations were generated rather than collected from operational pharmacy systems, this result should be interpreted as demonstrating SPC methodology rather than establishing the stability of real medication-use processes in Makkah and Jeddah; as reflected by the p-chart analysis. However, despite this stability the process operates at a relatively low sigma level (2.49), implying that a stable process does not necessarily imply acceptable performance. This distinction is consistent with quality management principles in which the stability of a process might not be associated with quality since there could be a significant number of defects within such process, which is centered at an undesirable mean performance level.

The calculated sigma level reflects the prevalence of defects within the generated dataset and therefore provides an illustration of Six Sigma calculations rather than an assessment of actual medication safety performance.

In Six Sigma quality management, higher sigma levels correspond to lower defect rates and greater process capability. Although healthcare processes rarely achieve the six-sigma benchmark, a sigma level below 3 generally reflects a relatively high frequency of defects that may expose patients to unnecessary safety risks. Therefore, while the process appears statistically stable, its current performance should not be considered clinically optimal.

The findings are broadly consistent with previous studies that reported measurable improvements in medication safety following the implementation of Six Sigma and Lean Six Sigma methodologies. Saudi hospital pharmacy studies have demonstrated reductions in dispensing errors, medication administration defects, and process waste following structured DMAIC interventions. Similarly, international studies have reported significant reductions in medication errors after implementing Lean Six Sigma, Failure Mode and Effects Analysis, and continuous quality monitoring systems. Unlike those intervention-based studies, however, the present study focused on illustrating SPC-based surveillance within a synthetic data environment rather than evaluating implemented quality-improvement initiatives.

The Pareto analysis identified wrong dose, omission, and wrong timing as the most prominent categories within the generated dataset. These categories are consistent with medication error types frequently reported in published medication safety literature. Nevertheless, because the synthetic dataset was generated using predefined input distributions, the observed frequencies should be interpreted as outputs of the simulation framework rather than direct evidence of actual error prevalence in hospitals within Makkah and Jeddah.

The Pareto analysis indicated that wrong dose, omission, and wrong timing were the most frequent medication error categories within the generated dataset. However, because the synthetic data generation process incorporated predefined probability distributions for the medication error categories, these findings should not be interpreted as empirical evidence regarding the prevalence of specific medication errors in Saudi hospital pharmacies. Instead, the results demonstrate how Pareto analysis can be used to prioritize improvement efforts when error-frequency data are available.

The Six Sigma analysis shows that there is a chance for process improvement in medication dispensing operations. Although the process is currently stable under statistical control, it does not meet high-quality performance standards. This highlights a critical gap between the process stability and clinical safety requirements, which is mainly important in high-risk healthcare environments such as hospital pharmacies.

The use of synthetic data generated through CTGAN allowed for structured process simulation and enabled the application of Statistical Process Control techniques in the absence of real patient-level data. While this approach supports methodological exploration, it also limits direct clinical generalization. While CTGAN enabled the generation of a structured dataset suitable for SPC analysis, the extent to which the synthetic records preserve complex real-world relationships among healthcare variables remains unknown and requires future validation using actual pharmacy datasets.

An important methodological limitation relates to the interpretation of the p-chart results. Because the observations were generated from a synthetic data model rather than collected through ongoing operational monitoring, the SPC chart cannot be interpreted as evidence that an actual medication-use process is under statistical control. The absence of out-of-control points primarily reflects the characteristics of the synthetic data generation process rather than verification of process stability in a real healthcare setting. Accordingly, the p-chart should be viewed as a demonstration of SPC methodology rather than a process-performance assessment.

Regarding implications, the current study reveals that the utilization of the Six Sigma approach along with SPC tools presents an effective methodological framework for examining medication errors. The methodological framework presented in this study may provide researchers and healthcare quality practitioners with an example of how SPC and DMAIC tools can be operationalized when only aggregated data are available. However, no direct recommendations for healthcare policy, operational performance, or patient-safety management should be inferred from the present findings without validation using real-world data.

Future research should focus on validating these findings using real-world hospital pharmacy data to confirm the observed patterns and assess the effectiveness of the proposed improvement strategies. Moreover, another interesting area of research would be the application of real-time monitoring approaches along with artificial intelligence tools for improving the prevention of drug dispensing errors.

## 5 Conclusion

This study explored the feasibility of applying Statistical Process Control and Six Sigma analytical tools within a synthetic-data environment derived from publicly available healthcare statistics.

The study further illustrated the use of p-charts, Pareto analysis, and cause-and-effect analysis to evaluate process performance and prioritize potential improvement opportunities. However, because the dataset was entirely synthetic, the reported error frequencies, defect rates, and sigma performance should be interpreted as methodological outcomes rather than direct measures of real-world hospital pharmacy performance.

Importantly, the Improve and Control phases presented in this study remain conceptual and were not implemented or empirically evaluated. Consequently, the effectiveness of the proposed interventions cannot be confirmed based on the current analysis.

Accordingly, the study should be viewed as a methodological proof-of-concept rather than an empirical assessment of medication dispensing performance in Saudi hospital pharmacies.

Despite these limitations, the study demonstrates how SPC and Six Sigma tools can support systematic monitoring, early detection of process variation, and data-driven quality management in pharmacy services. Future research should validate these findings using multicenter hospital pharmacy datasets, conduct prospective quality-improvement studies, evaluate the effectiveness of proposed interventions in routine clinical practice, and compare CTGAN-based synthetic data generation with alternative simulation and modeling approaches. Such studies would strengthen the evidence base for integrating SPC and Six Sigma methodologies into medication safety programs across Saudi healthcare organizations.

An additional limitation concerns the absence of external validation. Because no real patient-level medication-error dataset was available, it was not possible to compare the synthetic records against actual healthcare processes. Consequently, neither the validity of the generated data nor the generalizability of the resulting SPC and Six Sigma analyses can be empirically confirmed.

## Data Availability

The original contributions presented in the study are included in the article/Supplementary Material, further inquiries can be directed to the corresponding author.
