# Supplementary material for: Statistical process control within the Six Sigma DMAIC framework for monitoring medication dispensing errors in hospital pharmacies: a Saudi case study
Source: Front Pharmacol. 2026 Sep 29;17:1895605. doi: 10.3389/fphar.2026.1895605 (PMC13658443; doi:10.3389/fphar.2026.1895605)
Supplement: Supplementary file 1 [file Supplementaryfile1.docx]

**Title :Supplementary File S1. Detailed CTGAN Data Generation Workflow**

**1. Overview**

This study used a Conditional Tabular Generative Adversarial Network (CTGAN) to generate a synthetic patient-level dataset from aggregated healthcare statistics reported in the Ministry of Health (MOH) Statistical Yearbook 2024. The synthetic dataset was created solely for methodological demonstration of Statistical Process Control (SPC) and Six Sigma analysis and does not represent actual patient records.

**2. Workflow Steps**

**Step 1. Data Acquisition**

Aggregated healthcare indicators were extracted from the Saudi Ministry of Health Statistical Yearbook 2024, including:

- Hospital admissions
- Bed Occupancy Rate (BOR)
- Average Length of Stay (ALOS)
- Regional healthcare statistics
- Demographic indicators

**Step 2. Seed Dataset Construction**

A structured seed dataset consisting of 2,000 records was developed using:

- Regional distributions
- Population demographics
- Hospital utilization metrics
- Medication error assumptions
- ICD-10 disease-group proxies

**Step 3. Metadata Specification**

Variables were classified as:

**Categorical Variables**

- Region
- Health Cluster
- Age Group
- Gender
- Nationality
- Diagnosis Group
- Error Type

**Binary Variables**

- Has Error
- ER Wait >4h
- Readmitted Within 30 Days

**Continuous Variables**

- Bed Occupancy Rate
- Average Length of Stay
- Surgery Turnaround Time

**Step 4. Data Cleaning**

The following preprocessing steps were applied:

- Missing value handling
- Categorical encoding
- Boolean conversion to integer format
- Metadata validation

**Step 5. CTGAN Training**

The CTGAN model was trained using the cleaned seed dataset.

**Step 6. Synthetic Data Generation**

The trained model generated 10,000 synthetic observations.

**Step 7. Post-Generation Validation**

The generated dataset was evaluated through:

- Frequency comparisons
- Descriptive statistics
- Distribution comparisons
- Error prevalence assessment

**Step 8. SPC Analysis**

The final synthetic dataset was used to develop:

- P-Charts
- Pareto Charts
- Sigma-Level Calculations
- Fishbone Analysis
