# Supplementary material for: Statistical process control within the Six Sigma DMAIC framework for monitoring medication dispensing errors in hospital pharmacies: a Saudi case study
Source: Front Pharmacol. 2026 Sep 29;17:1895605. doi: 10.3389/fphar.2026.1895605 (PMC13658443; doi:10.3389/fphar.2026.1895605)
Supplement: Supplementary file 2 [file Supplementaryfile2.docx]

**Title : Supplementary File S2. Seed Dataset Construction Methodology**

**Dataset Size**

Total records: 2,000

**Variables Included**

**Demographic Variables**

| **Variable** | **Categories** |
| --- | --- |
| Gender | Male, Female |
| Nationality | Saudi, Non-Saudi |
| Age Group | 0-14, 15-24, 25-44, 45-64, 65+ |

**Geographic Variables**

| **Variable** | **Categories** |
| --- | --- |
| Region | Makkah, Jeddah |

**Clinical Variables**

| **Variable** | **Categories** |
| --- | --- |
| Diagnosis Group | 12 ICD-10 Proxy Categories |

**Operational Variables**

**Variable**

Bed Occupancy Rate

Average Length of Stay

Surgery Turnaround Time

**Medication Error Variables**

**Variable**

Has Error

Error Type

**Error Rate Assumptions**

Baseline medication error prevalence:

**5.8%**

Error categories:

| **Error Type** | **Probability** |
| --- | --- |
| Wrong Dose | 20% |
| Omission | 18% |
| Wrong Timing | 15% |
| Wrong Drug | 12% |
| Wrong Quantity | 10% |
| Wrong Patient | 8% |
| Mislabeling | 7% |
| Other | 10% |

These probabilities were selected to create a realistic demonstration dataset and do not represent observed rates from any hospital.

**Disease Distribution**

Twelve ICD-10 proxy groups were assigned according to national disease burden patterns.

**Population Distribution**

Age-group allocation was based on Saudi population pyramid estimates.
