# Supplementary material for: Statistical process control within the Six Sigma DMAIC framework for monitoring medication dispensing errors in hospital pharmacies: a Saudi case study
Source: Front Pharmacol. 2026 Sep 29;17:1895605. doi: 10.3389/fphar.2026.1895605 (PMC13658443; doi:10.3389/fphar.2026.1895605)
Supplement: Supplementary file 3 [file Supplementaryfile3.docx]

**Title :Supplementary File S3. CTGAN Training Configuration**

**Software Environment**

| **Item** | **Specification** |
| --- | --- |
| Programming Language | Python 3.11 |
| CTGAN Package | CTGAN 0.8.0 |
| Pandas | 2.2 |
| NumPy | 1.26 |

**Random Seed**

Random Seed = 42

**CTGAN Hyperparameters**

- Parameter (epoch, value = 300), number of full training passes. More = better quality but slower. 300 is a reasonable default for a 2000-row seed.
- Parameter ( batch_size, value = 500), How many rows the model sees per gradient update. 500 works well when seed = 2000.
- Parameter (generator_dim, value= 256) The generator neural network has 2 hidden layers, each 256 neurons wide. Bigger = more expressive but slower.
- Parameter (pac, value = 10) Packed AC-GAN groups 10 samples together before the Discriminator judges them. This is the key setting for handling rare categories like (Dispensing) without pac; rare categories collapse and disappear in the generated data.

| **Parameter** | **Value** |
| --- | --- |
| Epochs | 300 |
| Batch Size | 500 |
| Generator Dimensions | (256,256) |
| PAC | 10 |
| Embedding Dimension | 128 |
| Verbose | True |

**Training Dataset**

| **Metric** | **Value** |
| --- | --- |
| Training Rows | 2,000 |
| Generated Rows | 10,000 |
| Total Variables | 14 |

**Discrete Variables**

region

health_cluster

age_group

gender

nationality

diagnosis_group

has_error

error_type

er_wait_gt4h

readmitted_30

**Model Objective**

The model learned relationships among demographic, operational, and medication-error variables to generate statistically plausible synthetic observations.
