# Supplementary material for: Statistical process control within the Six Sigma DMAIC framework for monitoring medication dispensing errors in hospital pharmacies: a Saudi case study
Source: Front Pharmacol. 2026 Sep 29;17:1895605. doi: 10.3389/fphar.2026.1895605 (PMC13658443; doi:10.3389/fphar.2026.1895605)
Supplement: Supplementary file 4 [file Supplementaryfile4.docx]

**Title :Supplementary File S4. Python Scripts**

**Script 1. Seed Dataset Creation**

Purpose:

- Create 2,000-row seed dataset
- Apply demographic distributions
- Assign diagnosis groups
- Generate medication error categories

**Script 2. Data Cleaning**

Purpose:

- Handle missing values
- Validate metadata
- Convert binary variables

**Script 3. CTGAN Training**

Purpose:

- Define model
- Fit CTGAN model
- Save trained generator

**Script 4. Synthetic Data Generation**

Purpose:

- Generate 10,000 synthetic observations
- Export CSV dataset

**Script 5. SPC Analysis**

Purpose:

- Create ten subgroups
- Calculate proportions
- Compute control limits

**Script 6. Pareto Analysis**

Purpose:

- Count errors by category
- Rank frequencies
- Generate cumulative percentages

**Script 7. Sigma-Level Calculation**

Purpose:

Calculate:

- Defect Rate
- DPMO
- Sigma Level

**Script Repository Structure**

01_generate_seed.py

02_clean_data.py

03_train_ctgan.py

04_generate_synthetic.py

05_pchart.py

06_pareto.py

07_sigma_calculation.py

The scripts are provided to illustrate the analytical workflow used in this study. Researchers may adapt the code according to their software environment, CTGAN package version, and local data structure."
