# Supplementary material for: Statistical process control within the Six Sigma DMAIC framework for monitoring medication dispensing errors in hospital pharmacies: a Saudi case study
Source: Front Pharmacol. 2026 Sep 29;17:1895605. doi: 10.3389/fphar.2026.1895605 (PMC13658443; doi:10.3389/fphar.2026.1895605)
Supplement: Supplementary file 5 [file Supplementaryfile5.docx]

**Title :Supplementary File S5. Synthetic Data Fidelity Assessment**

**Objective**

Assess similarity between:

- Seed Dataset (2,000 records)
- Synthetic Dataset (10,000 records)

**Validation Metrics**

**Categorical Variables**

Evaluated using:

- Frequency comparison
- Percentage differences

**Continuous Variables**

Evaluated using:

- Mean comparison
- Standard deviation comparison
- Range comparison

**Error Prevalence Assessment**

| **Dataset** | **Error Rate** |
| --- | --- |
| Seed Dataset | 5.8% |
| Synthetic Dataset | 16.05% |

**Interpretation**

The generated dataset exhibited a higher prevalence of medication errors than the original seed dataset.

Possible explanations include:

- Minority-class oversampling
- Distributional drift
- Imperfect learning of class frequencies

**Impact on Sigma Level**

Because DPMO calculations depend directly on defect prevalence, the observed sigma level of 2.49 reflects synthetic dataset performance rather than actual healthcare system performance.

**Recommendation**

Future studies should evaluate synthetic data quality using:

- Jensen-Shannon Divergence
- Kullback-Leibler Divergence
- Kolmogorov-Smirnov Tests
- External validation using real hospital datasets

**Table S5.1. Distribution of medication error categories in the seed dataset design assumptions and generated synthetic dataset.**

| **Error Category** | **Intended Seed Distribution (%)** | **Expected Seed Errors (n≈116)*** | **Generated Synthetic Errors (n=1605)** | **Generated Distribution (%)** |
| --- | --- | --- | --- | --- |
| Wrong Dose | 20.0 | 23 | 316 | 19.69 |
| Omission | 18.0 | 21 | 295 | 18.38 |
| Wrong Timing | 15.0 | 17 | 239 | 14.89 |
| Wrong Drug | 12.0 | 14 | 193 | 12.02 |
| Wrong Quantity | 10.0 | 12 | 161 | 10.03 |
| Wrong Patient | 8.0 | 9 | 128 | 7.98 |
| Mislabeling | 7.0 | 8 | 113 | 7.04 |
| Other Errors | 10.0 | 12 | 160 | 9.97 |
| **Total** | **100** | **116** | **1605** | **100** |

*Expected seed errors were calculated from the target medication error prevalence of 5.8% applied to the 2,000-row seed dataset (2,000 × 0.058 = 116 errors). Values are approximate because the seed dataset was constructed from predefined probability distributions rather than observed patient-level records.

Interpre*ation of Table S5.1*

*The generated*synthetic dataset preserved the re*ative proportions of individual me*ication error categories reasonabl* well, with differences of less th*n 1 percentage point across all ca*egories when compared with the int*nded seed distribution. However, t*e overall prevalence of medication*errors increased from the target s*ed rate of approximately 5.8% to 1*.05% in the generated dataset.

Th*s finding suggests that the CTGAN odel was generally successful in peserving the conditional distribut*on of error types but did not full* preserve the marginal prevalence *f the medication-error class. The bserved inflation may reflect clas-balancing effects, sampling varia*ility, or distributional drift dur*ng synthetic data generation. Cons*quently, the reported defect rate,*DPMO, and sigma level should be in*erpreted as characteristics of the*generated synthetic dataset rather*than direct estimates of actual me*ication-error performance in hospi*al pharmacies.

Future studies sho*ld employ formal synthetic-data va*idation methods, including Jensen-*hannon divergence, Kullback-Leible* divergence, and external validati*n against real hospital datasets, *o further assess synthetic data fi*elity.

**Table S5.2. Overall Error Prevalence Comparison**

| **Metric** | **Seed Dataset** | **Synthetic Dataset** | **Relative Change** |
| --- | --- | --- | --- |
| Total Records | 2,000 | 10,000 | - |
| Error Rate (%) | 5.8 | 16.05 | +176.7% |
| Total Errors | 116 | 1,605 | - |
| Non-Error Records | 1,884 | 8,395 | - |
| Sigma Level Equivalent | Not Calculated | 2.49 | - |

**Interpretation**

Although category-level distributions were maintained, th* overall defect prevalence increas*ed substantially in the generated d*ataset. This discrepancy represents *limitation of the synthetic data* generation process and directly included the calculated DPMO and sigma level. Therefore, the sigma level reported in this study should be viewed as a methodological demonstration of SPC and Six Sigma analysis rather than an estimate of actual pharmacy performance.

.
