# Supplementary material for: Statistical process control within the Six Sigma DMAIC framework for monitoring medication dispensing errors in hospital pharmacies: a Saudi case study
Source: Front Pharmacol. 2026 Sep 29;17:1895605. doi: 10.3389/fphar.2026.1895605 (PMC13658443; doi:10.3389/fphar.2026.1895605)
Supplement: Supplementary file 6 [file Supplementaryfile6.docx]

**Title : Supplementary File S6**

**Synthetic Dataset Description**

**Dataset Size**

- Rows: 10,000
- Columns: 14

**Variables**

**Variable**

Region

Health Cluster

Age Group

Gender

Nationality

Diagnosis Group

BOR

ALOS

Surgery TAT

ER Wait >4h

Readmitted 30 Days

Has Error

Error Type

**Intended Use**

The dataset was generated exclusively for:

- SPC demonstrations
- Six Sigma calculations
- Healthcare quality methodology research

**Privacy Statement**

The dataset contains:

- No patient identifiers
- No clinical records
- No protected health information

**Important Note**

The dataset must not be interpreted as representing actual patient outcomes or actual medication-error rates in Makkah or Jeddah hospitals.
